# Neocortical Sox9+ radial glia generate glutamatergic neurons for all layers, but lack discernible evidence of early laminar fate restriction

**DOI:** 10.1186/s13064-017-0091-4

**Published:** 2017-08-16

**Authors:** E. S. Kaplan, K. A. Ramos-Laguna, A. B. Mihalas, R. A. M. Daza, R. F. Hevner

**Affiliations:** 1 Center for Integrative Brain Research, Seattle Children’s Research Institute, Seattle, WA 98101 USA; 2 Department of Neurological Surgery, University of Washington School of Medicine, Seattle, WA 98104 USA

**Keywords:** Sox9, Radial glial progenitors, Neural stem cells, Neurogenesis, Neocortex, Development

## Abstract

Glutamatergic neurons in the cerebral cortex are derived from embryonic neural stem cells known as radial glial progenitors (RGPs). Early RGPs, present at the onset of cortical neurogenesis, are classically thought to produce columnar clones of glutamatergic neurons spanning the cortical layers. Recently, however, it has been reported that a subset of early RGPs may undergo early commitment to upper layer neuron fates, thus bypassing genesis of deep layer neurons. However, the latter mode of early RGP differentiation was not confirmed in some other studies, and remains controversial. To further investigate the clonal output from early RGPs, we employed genetic lineage tracing driven by *Sox9*, a transcription factor gene that is expressed in all early RGPs. We found that early RGPs produced columnar clones spanning all cortical layers, with no evidence of significant laminar fate restriction. These data support the classic progressive restriction model of cortical neurogenesis, and suggest that early RGPs do not undergo early commitment to only upper or lower layer fates.

**Electronic supplementary material:**

The online version of this article (doi:10.1186/s13064-017-0091-4) contains supplementary material, which is available to authorized users.

## Introduction

The proper development of the cerebral cortex architecture is essential for numerous higher-order brain functions including sensory perception, language, and executive function. The vast majority of neurons in the cerebral cortex (~80%) are glutamatergic excitatory neurons, arranged into six horizontally oriented and histologically distinct layers [1]. Though not completely homogeneous, neurons within a layer commonly share characteristics including gene expression patterns, morphology, and connectivity [2]. The construction of this organized laminar circuitry involves a complex sequence of events whereby proliferative neural progenitors give rise to postmitotic neurons, which in turn migrate radially to their final positions. Initially, before neurogenesis begins (~E9-E10 in the mouse), neuroepithelial (NE) cells in the anterior neural tube proliferate to form a large pool of neural progenitors. These cells soon differentiate into more committed neural progenitors termed radial glial progenitors (RGPs). RGPs possess characteristic basal and apical processes that make contact with the pial and ventricular surfaces. As neurogenesis in the cortex begins (~E10.5), RGPs not only divide symmetrically to self-renew, but begin to divide asymmetrically to produce neurons, which in turn use RGPs’ processes as migratory guides. Most neurogenesis from RGPs occurs via the production of transit-amplifying intermediate progenitors (IPs), which in turn give rise to glutamatergic neurons [3, 4].

As neurogenesis in the neocortex progresses, neurons migrate radially away from their birthplace in the proliferative ventricular zone (VZ) or subventricular zone (SVZ), and towards the pial surface. Migrating neurons follow an “inside-out” pattern of lamination such that early born neurons populate the deeper layers of the cortical plate, while later born neurons migrate past them to progressively form more superficial layers [5, 6]. Therefore, birthdates of neurons are highly correlated with their ultimate position in the neocortical circuitry.

Though the correlation between the final positions of neurons and their birthdates is well-established, it remains unclear if all RGPs are equipotential and hence have the ability to produce neurons of all layers; or conversely, if specific progenitor populations are predetermined to give rise to neurons of particular layers [7]. One theory, the progressive restriction model, states that an RGP can generate projection neurons of all subtypes/ layers, and that the competence of RGPs to produce various neuron subtypes becomes progressively limited with developmental stage [8]. In support of this, transplantation experiments have shown that early progenitors transplanted into older cortex are capable of producing superficial layer neurons; however, later progenitors transplanted into younger cortex are incapable of producing deep layer neurons [8–10]. The progressive restriction model has also been supported by sparse labeling experiments and clonal analysis of progenitors, which have shown that RGPs in early cortex produce neurons bound for all layers [11–15]. Additionally, in vitro studies utilizing cultured progenitors has supported the idea that early progenitors are multipotent and that progenitor competency becomes progressively restricted [16].

In opposition to the progressive restriction model, some investigators have postulated the existence of RGPs that are inherently committed to produce neurons of a particular layer/subtype, irrespective of their developmental age. Interestingly, it has been noted that specific transcription factors expressed in some cortical neuron subtypes are also expressed earlier in development in subsets of progenitors, suggesting that classes of progenitors may already be committed to producing specific neuron subtypes at early stages [1]. One lineage tracing study reported that Cux2+ progenitors predominantly give rise to superficial layer neurons; even when labeling of these progenitors was accomplished in early cortex (E10.5) when deep layer neurons are typically produced [17]. However, subsequent studies investigating Cux2+ progenitors, as well as studies of other progenitor classes (Fezf2+ progenitors), have supported the progressive restriction model, and not the predetermined fate-restriction model [18–20]. Therefore, the existence of laminar fate-restricted RGPs remains highly controversial. Clearly, additional tools are needed to monitor neural progenitor lineages and provide evidence to support either the more classic progressive restriction model, or the predetermined fate-restriction model. In order to aid in the resolution of this controversy, we performed lineage tracing and clonal analysis of the progeny of RGPs in early cerebral cortex, using the transcription factor Sox9.

Sox9 is a member of the Sox (Sry HMG-box) family of transcription factors and is broadly expressed in NE cells throughout the VZ early in development [21, 22]. As cortical neurogenesis begins, Sox9 is known to be expressed in multipotent RGPs. Importantly, Sox9 has been shown to be critical for the regulation of both neuron and glia differentiation from RGPs [23–26]. By exploiting the *Sox9* gene as a marker of RGPs, we sought to clarify whether early Sox9-expressing (Sox9+) RGPs in the cortex are multipotent and contribute neurons to all layers, or if fate-restricted progeny can be observed. Here, we utilized inducible genetic fate mapping of cortical neurons using *Sox9* ^*CreER*^, which, when paired with the reporter gene *Ai14*, labels cohorts of Sox9-expressing RGPs and their progeny. Additionally, we were able to resolve the number and position of neurons derived from single Sox9+ RGPs by performing clonal analysis via the MADM (Mosaic Analysis with Double Markers) system [27, 28]. We find that Sox9-expressing neural stem cells give rise to neurons of all cortical layers, and find no evidence to support laminar fate-restricted RGP lineages.

## Methods

### Animals, tamoxifen administration, and tissue collection

**Fig. 1:**
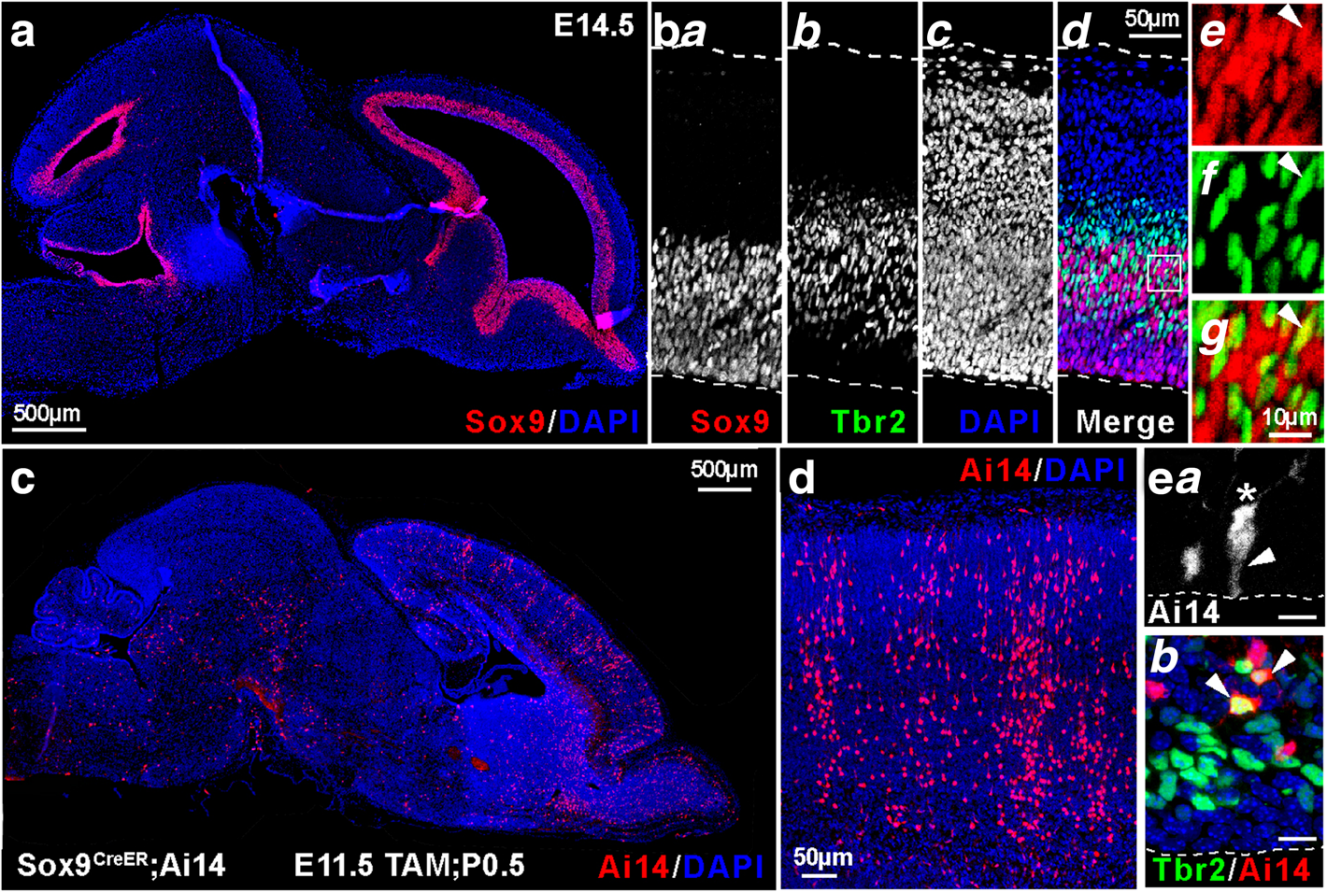
Sox9 is a Marker of Neural Progenitors. (**a**) Immuno-labeling of an E14.5 brain (parasagittal, rostral right, dorsal up) reveals Sox9+ (*red*) neural progenitors lining the ventricles. (**b**) E14.5 cortex immuno-labeled for Sox9 (*red*) and Tbr2 (*green*). (**b** ***a*** **-b** ***d***) Sox9+ neural progenitors, Tbr2+ intermediate progenitors (IPs), DAPI stained cell nuclei, and merged views. (**b** ***e*** **-b** ***g***) Higher magnification of ventricular zone/ subventricular zone region shows interspersed Sox9+ neural progenitors (*red*) and Tbr2+ IPs (*green*). Though predominantly non-overlapping, occasional Sox9+/ Tbr2+ co-labeling of cells was observed (*arrowhead*). (**c**) *Sox9* ^*CreER*^;*Ai14* tamoxifen-inducible reporter mice were utilized to label cohorts of Sox9+ progenitors and their progeny with red fluorescent protein variant tdTomato. P0.5 brain section shows tdTomato (Ai14) cell labeling resulting from tamoxifen injection of dam at E11.5. (E11.5Tam; P0.5) (**d**) Cortex from an E11.5 Tam; P0.5 pup shows progeny of Sox9+ neural progenitors give rise to columnar arrangement of cortical neurons. (**e** ***a***) *Sox9* ^*CreER*^;*Ai14* labels progenitors with morphology characteristic of radial glial progenitors (RGPs). Ai14+ cells from an E16.5Tam; P0.5 pup includes radial glial progenitor (RGP) with endfoot on ventricular interface (*arrowhead*), which appears to have recently divided to form a new cell with visible leading process (*asterisk*). Scale bar: 10 μm. (**e** ***b***) Ai14+ cells from E16.5Tam; P0.5 pup includes Tbr2+ IPs, as cells co-labeled for Ai14 (*red*) and Tbr2 (*green*) were observed (*arrowheads*). Scale bar: 10 μm

**Fig. 2:**
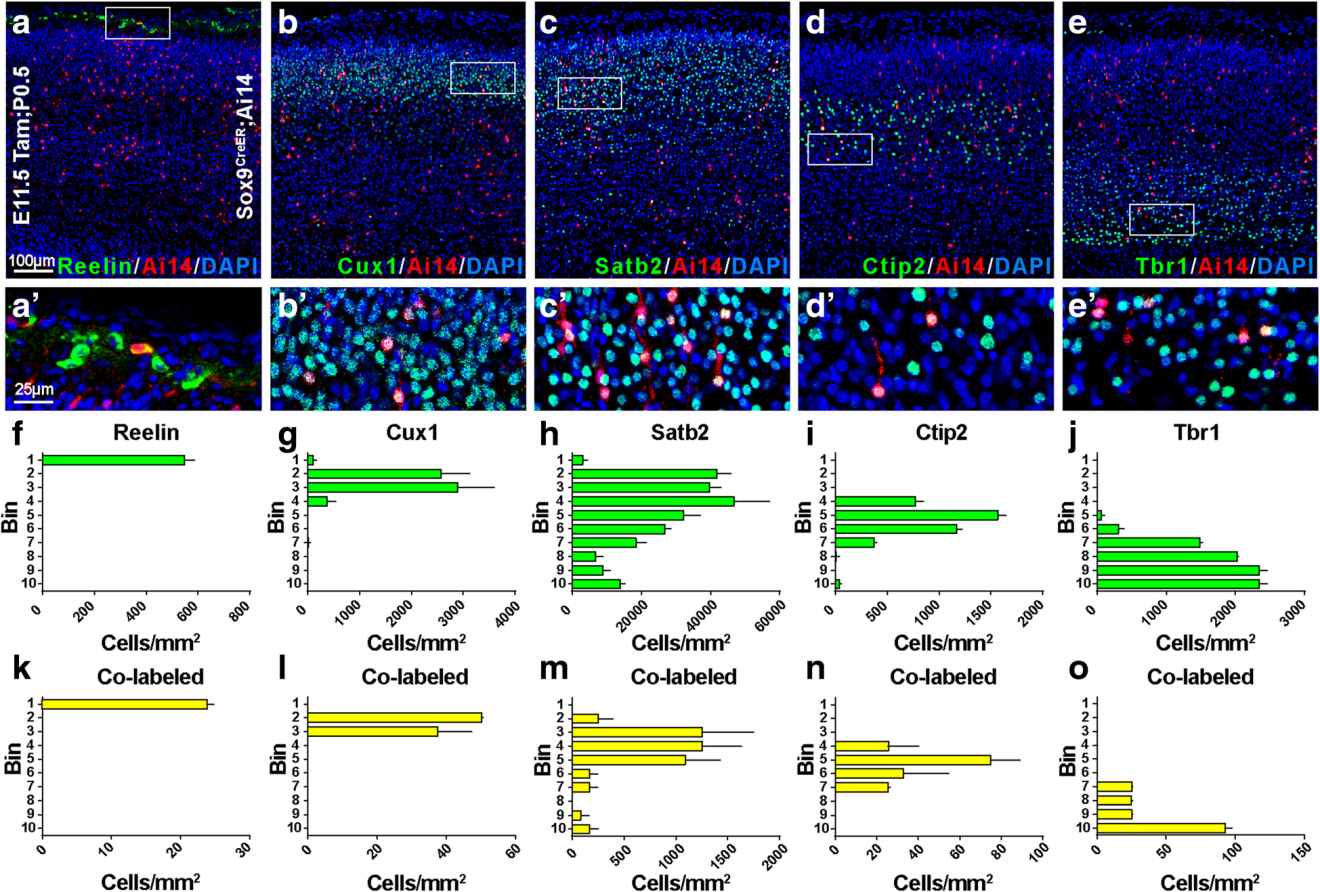
Sox9+ Neural Progenitors Give Rise to Neurons of all Cortical Layers. (**a**-**e**) Sox9+ neural progenitor cohorts and their progeny were labeled via tamoxifen injection of dams at E11.5, and pups were sacrificed at P0.5 (E11.5Tam; P0.5). Sox9+ progenitor-derived neurons were visible in all cortical layers and are shown with selected colocalized molecular markers in P0.5 cortex: (**a**) Reelin; (**b**) Cux1; (**c**) Satb2; (**d**) Ctip2; and (**e**) Tbr1. (**a’**-**e’**) Higher magnification views of boxed areas in (a-e) demonstrate colabeled cells (*yellow*). (**f**-**j**) Quantification of cortical immuno-labeling is shown as bin analysis for the number of cells positive for expression of each of the molecular markers in (a-e). Typically, bin 1 represents the marginal zone/ layer 1, bins 2–3 represent layers 2–4, bins 4–6 represent layer 5, bins 7–9 represent layer 6, and bin 10 represents the subplate. (**k**-**o**) Bin analysis of co-labeled cells (*yellow*) expressing both Ai14 (tdTomato, *red*) and each of the molecular markers (*green*)

**Fig. 3:**
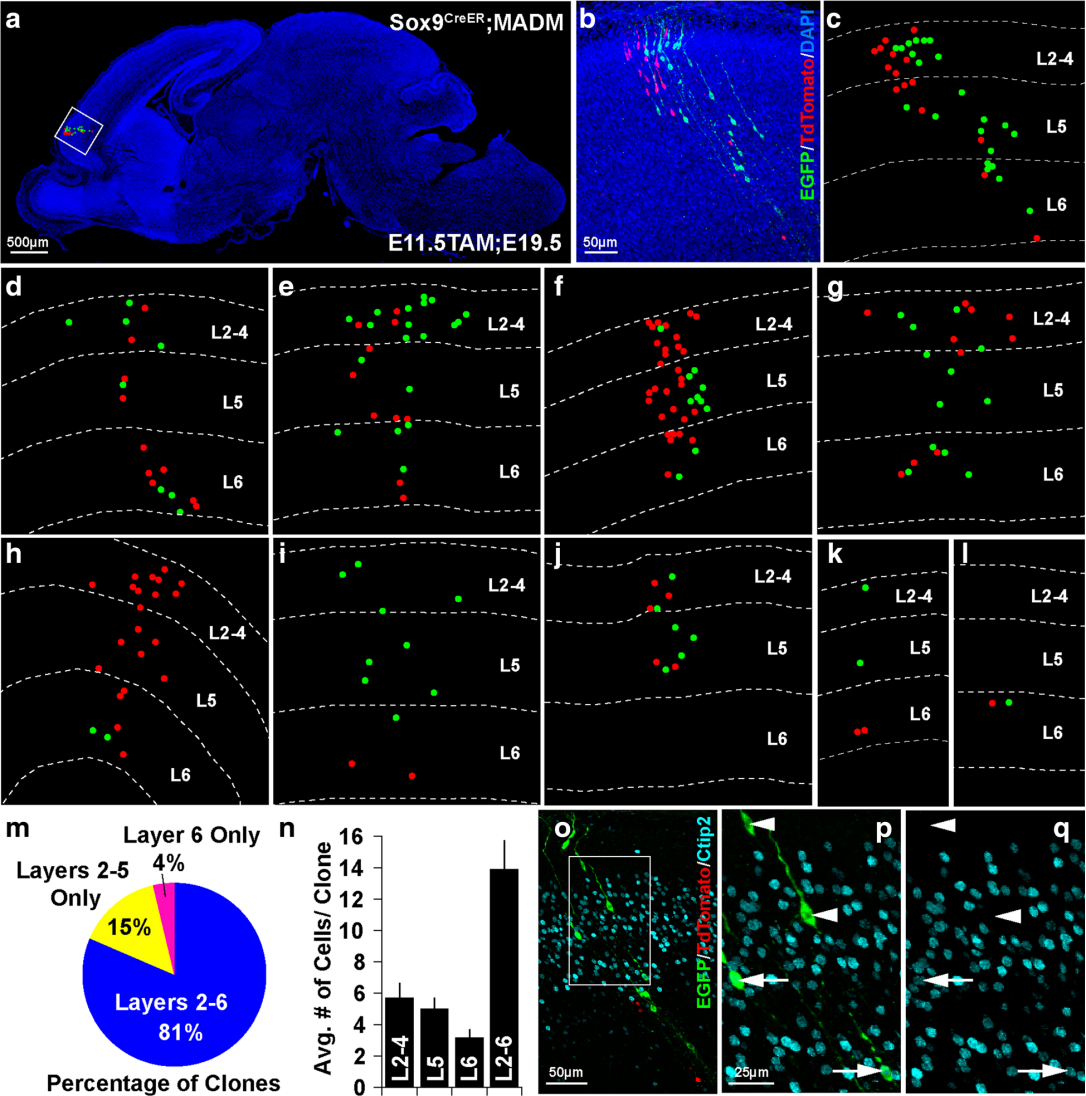
Individual Sox9+ Progenitors Produce Symmetric and Asymmetric Clones Spanning Cortical Layers. (**a**) By utilizing the *Sox9* ^*CreER*^;MADM system, tamoxifen injection at E11.5 yields sparse labeling of Sox9+ progenitors, which allows for clonal analysis of neurons derived from a single progenitor. Simplified dot representation of Sox9+ progenitor-derived clone overlaid onto sagittal section of E19.5 brain, reveals the cortical clone’s location. (**b**) Magnification of inset area displays example Sox9+ progenitor-derived cortical clone, comprised of EGFP (*green*) and tdTomato (*red*) expressing neurons. (**c**) Identical clone is portrayed in simplified dot representation to resolve cell position in relation to cortical lamina. (**d**-**l**) Representations of Sox9+ progenitor-derived clones demonstrate the size and laminar spread of neurons observed for each of these clones. Note certain clones display relatively equal numbers of EGFP and tdTomato expressing neurons implying a symmetric pattern of cell division, while other clones show proportions that imply asymmetric division. (**m**) Each clone was analyzed in order to assess number of neurons present in superficial layers (2–4), deep layer 5, and deep layer 6 (as no neurons were observed in layer 1). Pie chart displays the percentage of clones in which neurons were observed in cortical layers 2–6, layers 2–5 only (no neurons in layer 6), and layer 6 only (no neurons in layers 2–4 or layer 5). (**n**) Histogram displays the average number of neurons observed in each clone throughout layers 2–6, as well as within superficial layers 2–4, deep layer 5, and deep layer 6. (**o**) E19.5 brain section, including Sox9+ progenitor-derived clone (EGFP, *green*; tdTomato, *red*), immuno-labeled for Ctip2 (cyan), a layer 5 neuronal marker. (**p**-**q**) Magnification of inset area in o demonstrates molecular heterogeneity of neurons within a single Sox9+ progenitor-derived clone. A number of the neurons within the clone express Ctip2 (*arrows*, *bottom*), while others do not (*arrowheads, top*)

Mice were kept in a 12 h light/dark cycle, with food and water ad libitum. All animal experimental procedures were performed with Institutional Animal Care and Use Committee approval. Lineage tracing experiments (Figs. 1 and 2) utilized *Sox9* ^*CreER*^ ^T2^ mice (RIKEN, RBRC #05522) bred to *Ai14* reporter mice (JAX Stock 007914). Clonal analysis experiments (Fig. 3 and Additional file 1: Figure S1) utilized *MADM-11* ^*GT*^ (JAX Stock 013749) and *MADM-11* ^*TG*^ (JAX Stock 013751) mice. For MADM labeling, *Sox9* ^*CreER*^ ^*T2+/−*^; *MADM-11* ^*GT/GT*^ mice were bred to *MADM-11* ^*TG/TG*^ mice. Time of pregnancy was determined by the presence of a vaginal plug in the morning hours (E0.5). For lineage tracing and clonal analysis, pregnant females were injected intraperitoneally with tamoxifen (Sigma) dissolved in corn oil (Spectrum). Tamoxifen was administered at 10 mg/kg for Ai14 labeling experiments and 100 mg/kg for MADM labeling experiments. For Ai14 labeling experiments, P0.5 pups were decapitated and brains were fixed in 4% paraformaldehyde in 0.1 M sodium phosphate buffer at 4 °C for 4 h. Fixed brains were cryo-protected in 30% sucrose, frozen in optimum cutting temperature compound (OCT, Tissue-tek), and sectioned on a cryostat (Leica) at 12 μm. A similar procedure was completed for MADM clonal analysis labeling experiments, with the exception that due to the occurrence of high dose tamoxifen-induced dystocia, embryos were recovered at E19.5 from pregnant dams. For MADM labeling experiments, serial section examination was required; therefore, cryosections were cut at 30 μm to simplify and expedite confocal microscope analysis. In order to analyze early Sox9+ progenitor derived clones at postnatal ages with the MADM system, caesarian section was performed at E19.5 on pregnant females (previously tamoxifen injected at E11.5). Pups removed from the female were cleaned/stimulated to breathe by gentle rubbing with gauze moistened with warm water on a recirculating water heating pad. Pups, now considered P0, were transferred to a lactating foster mother. At P7, pups were perfused intracardially with saline followed by 4% paraformaldehyde in 0.1 M sodium phosphate buffer. Fixed brains were cryo-protected and sectioned as described above.

### Immunohistochemistry

Immunohistochemistry was performed as previously described [29]. The following primary antibodies were utilized at the indicated dilutions: rat anti-Ctip2 (1:1000; Abcam), rabbit anti-Cux1 (1:1000; Santa Cruz Biotechnology), rabbit anti-dsRed (1:1000; Clontech), chicken anti-GFP (1:500; Abcam), mouse anti-Reelin (1:1000; Calbiochem), mouse anti-Satb2 (1:400; Abcam), rabbit anti-Sox9 (1:500; EMD Millipore), rabbit anti-Tbr1 (1:2000; RFH laboratory), rat anti-Tbr2 (1:250 eBioscience). Fluorescently tagged secondary antibodies utilized: Alexa Fluor −488, −568, −647 (1:400; Molecular Probes).

### Image acquisition and analysis

Single-plane optical sections and stacks were acquired with Zeiss LSM-710 confocal microscope. Low magnification images of whole brain sections were acquired with Zeiss AxioImager.Z1 or Olympus VS120 Virtual Slide System. Images were adjusted for brightness and contrast in Adobe Photoshop. For image analysis, grids with 10 equidistant rows (bins) were overlaid onto images extending from the top of the pia surface to the bottom of the cortical plate. For Ai14 labeling experiments, cells were counted using the cell counter function in ImageJ (NIH), and reported as density per area (mm^2^) within bins. Typically at the ages analyzed (E19.5-P0.5): bin 1 represents the marginal zone/layer 1, bins 2–3 represent layers 2–4, bins 4–6 represent layer 5, bins 7–9 represent layer 6, and bin 10 represents the subplate. For P7 tissue, bin 1 represents the marginal zone/layer 1, bins 2–4 represent layers 2–4, bins 5–6 represent layer 5, bins 7–9 represent layer 6, and bin 10 represents the subplate. Data for the Ai14 labeling experiments was analyzed from 2 to 3 sections from 2 to 3 brains, per condition. For MADM labeling experiments, data was analyzed and reported by manual counting of cells within 10 equidistant bins (as described above). Bin counts were collapsed and data reported as cell position within cortical layers 2–4, layer 5, or layer 6. For MADM labeling experiments only red and green cells were counted, double-labeled (yellow) cells were omitted. Data from MADM labeling experiments analyzed at E19.5 was derived from 27 clones observed within 5 brains (3–9 clones recorded per brain).

## Results

### Sox9 is a marker of radial glial progenitors

To confirm that Sox9 is expressed in RGPs and not IPs, immunohistochemistry was performed on wild type (WT) E14 tissue (Fig. 1a), which revealed labeling characteristic of neural progenitors, lining the ventricles. Immunofluorescence of Sox9 and Tbr2 proteins showed Sox9+ cells in the VZ, and Tbr2+ intermediate progenitors (IPs) in the VZ and SVZ of the developing cortex (Fig. 1b*a-d*). Importantly, although areas of the developing cortex showed interspersed Sox9 and Tbr2 labeling, most cells expressed one marker or the other. A few cells appeared to express both Sox9 and Tbr2 (Fig. 1b*e-g*). Such overlap likely reflects a transient condition where differentiation of RGPs to IPs is in progress.

In order to address if early Sox9+ RGPs give rise to neurons of all layers, or rather to neurons of a specific layer or subtype, we initially utilized *Sox9* ^*CreER*^ *;Ai14* mice. These mice enabled temporally controlled, tamoxifen inducible, stable fluorescent labeling of cohorts of Sox9+ progenitors and their lineages. *Sox9* ^*CreER*^;*Ai14* dams were injected with tamoxifen at E11.5 and offspring were sacrificed at P0.5 (E11.5Tam; P0.5). Brain sections revealed Ai14+ (red fluorescent) cells throughout the cortex and other brain areas, identifying daughter cells of Sox9+ progenitors (Fig. 1c). Characteristic columnar organization of Ai14+ neurons in the cortex was visible in E11.5 Tam; P0.5 brain sections (Fig. 1d), and revealed that neurons derived from Sox9+ progenitors were positioned throughout the cortical layers. Imaging of sections from E16.5 Tam; P0.5 brains confirmed that Sox9+ neural progenitors are in fact RGPs with characteristic morphology (Fig. 1Ea). Additional immuno-labeling of E16.5 Tam; P0.5 brains confirmed that Sox9+ RGPs give rise to Tbr2+ IPs (Fig. 1e*b*), in addition to neurons.

### Early Sox9+ RGPs give rise to neurons of all cortical layers

Though the progeny of Sox9+ RGPs induced at E11.5 appeared to span all layers of cortex (Fig. 1d), we sought to determine if these Ai14+ cells represented various neuronal subclasses via immuno-labelling for layer-specific markers. Tissue from P0.5 pups, which had received tamoxifen at E11.5, revealed that Ai14+ neuronal progeny of Sox9+ RGPs expressed several laminar markers (Fig. 2). We observed Ai14+ neurons which expressed Reelin (Fig. 2a, *a’*), Cux1 (Fig. 2b, *b'*), Satb2 (Fig. 2c, *c’*), Ctip2 (Fig. 2d, *d’*), and Tbr1 (Fig. 2e, *e’*). Bin analysis revealed the distribution of cells expressing these molecular markers throughout the cortical layers (Fig. 2f-j). We also performed bin analysis to quantify co-labeled cells, which expressed both Ai14 (tdTomato; red) and the molecular marker of interest (Reelin, Cux1, Satb2, Ctip2, or Tbr1; green) (Fig. 2k-o). These results provide evidence that early Sox9+ RGPs are capable of differentiating into neurons which express a diversity of molecular markers characteristic of various cortical layers and glutamatergic neuron subtypes.

### Clonal analysis of individual Sox9+ neural progenitor lineages reveals symmetric and asymmetric clones spanning cortical layers

Since lineage tracing with *Sox9* ^*CreER*^ *;Ai14* produced extensive labeling in cortex with potential clonal overlap, further studies were necessary to verify the clonal output of individual RGPs. In order to conduct clonal analysis and better resolve the laminar positions of the neuronal progeny from individual Sox9+ RGPs, we utilized the MADM (Mosaic Analysis with Double Markers) system [28]. The MADM system allows dividing progenitors to reconstitute and express one of two fluorescent markers (EGFP, or tdTomato) in each of their daughter cells. If these daughter cells divide further, the expression of the fluorescent marker is also stably expressed in their progeny, allowing for visualization of all clonal descendants. The MADM system accomplishes this via Cre-recombinase-mediated interchromosomal recombination during the G_2_ phase of the cell cycle in dividing cells, followed by X-segregation (G_2_-X, segregation of recombinant sister chromatids into separate daughter cells). G_2_-X MADM events result in distinct and stable labeling of the two daughter cells and their lineages. This system allows for an assessment of the pattern of cell division (symmetric vs. asymmetric) and the ability to assay the overall potential of the progenitor (total number of red and green neurons derived from a progenitor). Conversely, this system can also result in labeling of one daughter cell with both EGFP and tdTomato, and no fluorophore labeling in the other daughter cell in the event of G_2_-Z segregation (congregation of recombinant sister chromatids in the same daughter cell). Additionally, recombination events occurring in the G_1_ or G_0_ phases of the cell cycle can also result in simultaneous reconstitution of EGFP and tdTomato within one cell. These circumstances which result in double-labeled (yellow) cells do not allow for reliable quantitative clonal analysis. Therefore, yellow double-labeled cells found within our experiments were not quantified. Fluorescently labeled red and green cells were analyzed to assess the size and laminar localization characteristics of Sox9+ progenitor-derived clones.

In order to label early Sox9+ RGPs and their descendants sparsely and in a temporally restricted manner, *Sox9* ^*CreER*^ mice were bred into the MADM system. Tamoxifen (Tam) was administered to E11.5 timed-pregnant mice. The dose of tamoxifen was titrated to a level that ensured only a small number of clones would be labeled within the cortex to allow for clear and accurate assessment of neurons derived from single Sox9+ RGPs. E19.5 brains (harvested before birth to avoid dystocia, a complication of tamoxifen) were fixed, and serial sections of brain were analyzed to assess the size of, and distribution of cells within Sox9+ RGP-derived clones in the cortex (Fig. 3). A representative clone residing in the frontal cortex (Fig. 3a-c) shows characteristic columnar structure of neurons present throughout the cortical layers. Confocal fluorescent images of clones were analyzed for number and distribution of neurons. For presentation, the position of red and green labeled neurons is shown as dots with demarcations for superficial layers 2–4, deep layer 5, and deep layer 6 (Fig. 3c). Although the total number of neurons in each clone varied, several of the clones contained abundant numbers of both red and green cells (Fig. 3d-g), consistent with a symmetric division of the original Sox9+ RGP into two daughter RGPs. This renewing division creates two new RGPs, which retain considerable proliferative capacity. We observed some clones which consisted of only green or only red cells. In this case, it is assumed that one of the original daughter cells was eliminated via apoptosis. Some clones observed were consistent with an asymmetric pattern of cell division in which the original RGP divided to produce one RGP, which retained high proliferative capacity, and one neuron or intermediate progenitor (IP, Fig. 3h-i). The pattern of neurons observed for one small clone was consistent with differentiation of the Sox9+ RGP into two IPs, which in turn each differentiated into two neurons (Fig. 3k).

With an aim to pursue evidence of laminar fate-restricted clones, we analyzed the laminar position of Sox9+ RGP-derived neurons within individual clones. 81% of clones observed (22/27 clones from *n* = 5 brains) contained neurons dispersed throughout the superficial layers 2–4, and the deep layers 5 and 6 (layers 2–6, Fig. 3m). 15% of clones (4/27 clones from *n* = 5 brains) contained neurons in the superficial layers and in deep layer 5, but not in layer 6 (layers 2–5 only, Fig. 3j and m). Finally, a single clone (1/27 clones from *n* = 5 brains) contained two neurons (one red and one green) residing only within deep layer 6 (layer 6 only, Fig. 3l and m). This final clone is consistent with a terminal symmetric differentiation of the Sox9+ RGP into two neurons. Besides this final clone consisting of two neurons, we did not observe other clones where neurons were restricted to a particular lamina. In fact, all other clones (96%) contained neurons which spanned both deep and superficial layers. Interestingly, this even extended to clones containing relatively few neurons (Fig. 3k). We analyzed the average number of cells contained within each clone and how the distribution of those cells mapped to the superficial and deep layers. The average number of cells per clone found within all the cortical layers (L2–6) was 13.89 ± 1.85 (no neurons were observed in layer 1, *n* = 27 clones from 5 brains). We also analyzed the average numbers of cells present in layers 2–4 (5.7 ± 0.94), layer 5 (5.0 ± 0.68), and layer 6 (3.2 ± 0.53, *n* = 27 clones from 5 brains).

Since the vast majority of clones we observed contained neurons spanning the deep and superficial layers of the cortex, we sought to confirm that these neurons were distinct not only in laminar position but in molecular expression. E19.5 tissue from *Sox9* ^*CreER*^;MADM mice containing clones induced at E11.5 (E11.5Tam; E19.5) was immuno-labeled for Ctip2, a protein expressed predominantly in layer 5 neurons. Indeed, neurons within a single Sox9+ RGP-derived clone contained both Ctip2+ and Ctip2- neurons (Fig. 3o-q). Although the majority of deep-layer neurons have completed migration at the developmental period analyzed (E19.5), outward migration of late-born, upper-layer neurons continues through postnatal day 7 [30]. Such postnatal migrations could potentially hinder our ability to resolve final neuronal positions.

High dose tamoxifen administration required for sparse labeling of clones using the MADM system causes dystocia, making postnatal analysis of clones highly challenging. However, by fostering mouse pups we were able to successfully analyze a cortical clone from postnatal day 7 (P7) tissue (Additional file 1: Figure S1). This clone consisted of neurons which spanned the cortical layers (layers 2–6), consistent with our E19.5 analysis (Fig. 3). Therefore, analysis of Sox9+ RGP-derived clones revealed a pattern consistent with the classic model of cortical development in which early RGPs generate principal neurons of all subtypes/layers. We did not observe any evidence of laminar fate-restricted clones stemming from early Sox9+ RGPs.

## Discussion

The cerebral cortex is involved in several key cognitive functions. Proper development of the cortex relies on progenitors’ production of neurons, which are then organized into six horizontally oriented layers. Glutamatergic projection neurons are the predominant neuron type making up the cortex, and are derived from RGPs and IPs located at or near the ventricular surface. New neurons migrate away from proliferative areas and into the cortical plate. Early born neurons form the deep layers, while later born neurons migrate past these neurons to form the superficial layers. This inside-out pattern of cerebral cortex development is well documented, and the result is an elaborate six layer structure containing several projection neuron types [5, 6].

Although cortical projection neurons are quite heterogeneous, neurons found within the same layer do tend to share characteristics such as gene expression patterns and axonal targets. A key question related to the heterogeneous projection neuron types found throughout the cortical plate is whether or not the proliferative RGPs which spawn them are themselves a homogeneous group.

Significant previous evidence indicates that most or all early RGPs can produce diverse neurons for all cortical layers; and, that as development advances, RGPs progressively lose the ability to produce deep layer neurons. This idea, termed the progressive restriction model, is supported by several experimental avenues. The timing-dependent nature of RGP capacity has been shown via transplantation experiments demonstrating that early progenitors are able to give rise to neurons of any layer; while later progenitors are restricted to producing upper layer neurons, even when exposed to an early environment [8–10]. This model has also been supported by *ex vivo* culturing of progenitors derived at different developmental stages [16]. Additionally, studies using lineage tracing of progenitors have shown that early progenitors can give rise to neurons present throughout the cortical layers [11, 12, 15].

Together, these studies support the idea that early in development, cortical RGPs are multipotent and able to give rise to projection neurons of all subtypes. The ability of these RGPs to produce heterogeneous cortical neurons (deep or superficial layer; callosal, subcerebral, or corticothalamic projecting etc.) may primarily depend on developmental stage. Accordingly, a majority of fate specification decisions in early cortex may be resolved by the cortical environment on the cell’s birthday [31]. While much data indicates that early RGPs in aggregate can produce neurons destined for all cortical layers, relatively few studies have demonstrated laminar multipotency of single RGPs [1, 11, 32].

Another model of fate restriction from early RGPs, termed the predetermined fate-restriction model, has been recently proposed [1, 7]. This model is based on evidence that a portion of RGPs are lineage-committed, and thus predetermined to create certain subtypes of projection neurons destined for particular layers. The predetermined fate-restriction model also seems to correlate with the molecular heterogeneity of RGPs, and their relation to the heterogeneous molecular expression profiles of postmitotic neurons. For example, transcription factors Cux2 and Fezf2 appear to be expressed in subsets of progenitors, and in subsets of postmitotic neurons found predominantly in superficial (Cux2) or deep (Fezf2) cortical layers [1].

Recently, evidence for the existence of lineage-committed progenitors was found by using tamoxifen induced fate-mapping of Cux2-expressing RGPs [17]. It was shown that early (E10.5) Cux2-expressing RGPs produced neurons found predominantly in the superficial layers. It was posited that the Cux2-expressing RGPs mainly undergo regenerative proliferative division early in development when deep layer neurons are born, then later switch to neurogenic division to generate superficial neurons at the appropriate time.

However, a different study reached different conclusions, finding that early Cux2-expressing RGPs produced neurons which migrated to all cortical layers, with equal percentages of these neurons expressing laminar markers Tbr1, Ctip2, or Cux1. In addition, many of the Cux2-labeled cells in the proliferative zones were identified as migrating interneurons. And, Cux2 lineage tracing was found to label not only progenitor cells but also postmitotic neurons; complicating the interpretation. Moreover, clones derived from early Fezf2-expressing RGPs gave rise to neurons of all cortical layers [18]. The debate on the fate-restricted nature of Cux2-expressing RGPs remains complicated and unresolved [18, 19]. In any case, Cux2-expressing RGPs appear to be a controversial candidate to test the progressive restriction model. Importantly, this controversy highlights the need for additional tools to probe for the existence of fate-restricted pools of cortical RGPs.

We utilized Sox9 as a novel RGP marker in order to conduct genetic lineage-tracing and clonal analysis of early progenitors, and thereby pursue evidence of laminar fate-restricted progenitors. Sox9 has been shown to be important in the induction and maintenance of neural stem cells, and is expressed in early cortical RGPs [22, 25, 26].

Here, we further examined the specificity of Sox9 expression in the ventricular zone of early cortex. Our results demonstrated that Sox9 is very specific for RGPs (Fig. 1), indeed more specific than Pax6, which is co-expressed with Tbr2 in a subset (~26%) of IPs [29]. We utilized tamoxifen inducible *Sox9* ^*CreER*^;*Ai14* mice to show that recombination at E11.5 resulted in labeling of neurons throughout the cortical layers. These lineages of Sox9+ RGPs included neurons that expressed diverse laminar markers: Reelin, Cux1, Satb2, Ctip2, and Tbr1 (Fig. 2). Lastly, we paired *Sox9* ^*CreER*^ and MADM to analyze the laminar fates of neurons derived from single Sox9+ RGPs (Fig. 3). Using *Sox9* ^*CreER*^;MADM mice, we observed that clones induced on E11.5 gave rise to both symmetric and asymmetric clones, extending across the cortical layers. Although clones derived from Sox9+ RGPs varied considerably in size, the distribution of cells within clones was similar, in that the vast majority possessed neurons in both deep and superficial layers. Only one clone, consisting of two neurons, was constrained to a single layer (layer 6), which could result from early terminal differentiation of the RGP into two neurons. We did not observe clones where a substantial number of neurons were restricted to a particular lamina; or even a deep neuron-only or superficial neuron-only clone containing more than 2 cells.

Interestingly, our findings also demonstrate a range of proliferative capacity of early RGPs. We observed a continuum of clone sizes including very small (2 neurons) and very large (>35 neurons) clones. We also observed lopsided asymmetric clones in which the initial division of the Sox9+ RGP presumably gave rise to an IP, and a RGP retaining high proliferative capacity (Fig. 3). Our observations, including the average number of neurons per clone (~14) and the occurrence of symmetric and asymmetric clones at the E11.5 induction time point, are consistent with previously reported MADM clonal analysis using *Emx1* ^*CreER*^ mice [11]. Importantly, that study also supported the classic progressive restriction model, and found no evidence of laminar fate-restricted progenitors when labeling clones via *Emx1* ^*CreER*^-MADM mice [11]. We also observed both Ctip2+ and Ctip2- neurons within a single clone, demonstrating gene expression diversity within Sox9+ RGP-derived clones. Although our findings cannot rule out the existence of fate-restricted progenitors as a small minority of RGPs, we demonstrate here that Sox9+ RGPs give rise to neurons of all cortical layers, in accordance with the classic progressive restriction model.

The neocortex contains several types of glutamatergic projection neurons that differ in key properties including laminar position, structural morphology, electrophysiology, gene expression, and axonal targets. Some evidence suggests that the generation of this exquisite diversity may be controlled via the dynamic expression of several genes that alter fate decisions in young neurons [7]. Although we cannot dismiss the possibility that fate-restricted progenitors exist, it is conceivable that a relatively homogeneous group of RGPs gives rise to immature neurons which then undergo dynamic changes in gene expression to lock in their mature properties. Importantly, it is known that for a limited time new postmitotic neurons co-express several transcription factors, which later become restricted to a particular projection neuron subtype [1]. Between E12.5-E14.5 for instance, some neurons co-express high levels of Ctip2 and Tbr1, before ultimately differentiating to subcerebral and corticothalamic projection neurons [33, 34]. Similarly, at E13.5 deep layer neurons co-express Ctip2 and Satb2 before diverging into subcerebral and callosal projection neurons [35, 36]. The relative expression of several genes including Sox5, Tbr1, Fezf2, Ctip2, and Satb2 may govern the fate decisions of new projection neurons. Studies have found that altering expression of these critical genes results in a dramatic shift in the relative numbers of callosal, subcerebral, and corticothalamic projection neurons in the cortex [1, 37]. Thus, the remarkably diverse nature of cortical projection neurons may result from fate decisions guided by relative expression of several key genes at the postmitotic stage, with initial fate decisions in progenitors remaining modifiable as new sets of molecular patterns are instituted during differentiation from RGP to IP to neuron [37, 38].

## Additional file

Additional file 1: Figure S1. Early Sox9+ Progenitor-derived Clone Analyzed on Postnatal Day 7 (P7) Spans Cortical Layers. (A) By utilizing the *Sox9* ^*CreER*^;MADM system, tamoxifen injection at E11.5 yields sparse labeling of Sox9+ progenitors, which allows for clonal analysis of neurons derived from a single progenitor. Simplified dot representation of Sox9+ progenitor-derived clone overlaid onto sagittal section of P7 brain, reveals the cortical clone’s location. (B) Magnification of inset area displays Sox9+ progenitor-derived cortical clone from P7 brain, comprised of tdTomato (red) expressing neurons. (C-D) Higher magnification images of neurons within this clone demonstrate neuronal morphological maturity at P7. (E) Identical clone is portrayed in simplified dot representation to resolve cell position in relation to cortical lamina. Neurons comprising the clone span the cortical layers, which is consistent with clones analyzed at E19.5. (JPEG 1442 kb)
