# Supplementary figures and images for: Neocortical Sox9+ radial glia generate glutamatergic neurons for all layers, but lack discernible evidence of early laminar fate restriction
Source: Neural Dev. 2017 Aug 16;12:14. doi: 10.1186/s13064-017-0091-4 (PMC5559824; doi:10.1186/s13064-017-0091-4)

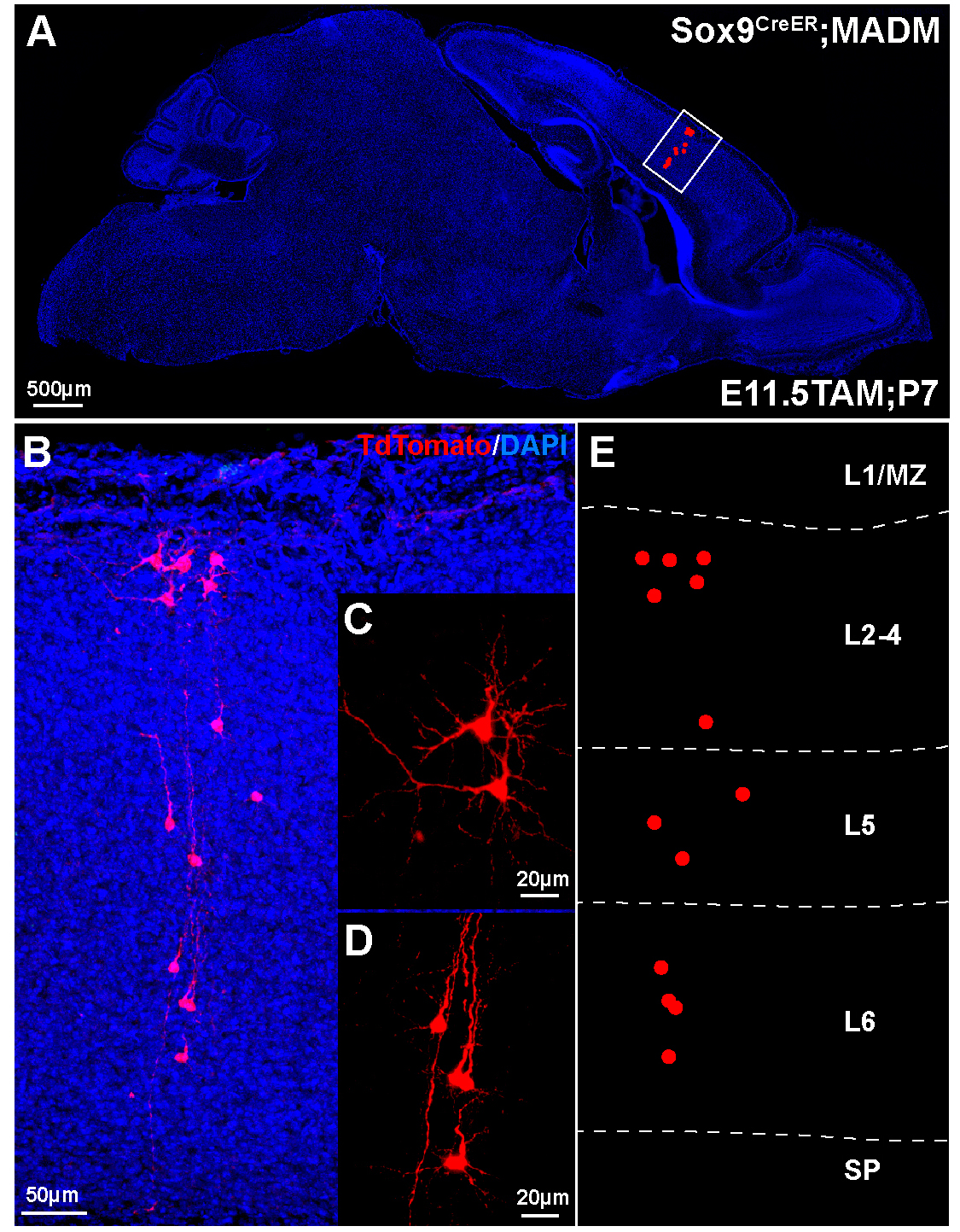

Supplement: Additional file 1: Figure S1. — Early Sox9+ Progenitor-derived Clone Analyzed on Postnatal Day 7 (P7) Spans Cortical Layers. (A) By utilizing the Sox9 CreER;MADM system, tamoxifen injection at E11.5 yields sparse labeling of Sox9+ progenitors, which allows for clonal analysis of neurons derived from a single progenitor. Simplified dot representation of Sox9+ progenitor-derived clone overlaid onto sagittal section of P7 brain, reveals the cortical clone’s location. (B) Magnification of inset area displays Sox9+ progenitor-derived cortical clone from P7 brain, comprised of tdTomato (red) expressing neurons. (C-D) Higher magnification images of neurons within this clone demonstrate neuronal morphological maturity at P7. (E) Identical clone is portrayed in simplified dot representation to resolve cell position in relation to cortical lamina. Neurons comprising the clone span the cortical layers, which is consistent with clones analyzed at E19.5. (JPEG 1442 kb) [file 13064_2017_91_MOESM1_ESM.jpg]
